# Abdominoinguinal approach in en bloc resection of retroperitoneal sarcoma involving iliac vessels with graft interposition

**DOI:** 10.3389/fonc.2022.1040833

**Published:** 2022-12-23

**Authors:** Ang Lv, Bo-Nan Liu, Dao-Ning Liu, Zhen Wang, Chun-Yi Hao

**Affiliations:** Key Laboratory of Carcinogenesis and Translational Research (Ministry of Education/Beijing), Sarcoma Center, Peking University Cancer Hospital & Institute, Beijing, China

**Keywords:** retroperitoneal sarcoma, en bloc resection, abdominoinguinal approach, iliac vessels, graft interposition

## Abstract

**Background:**

Retroperitoneal sarcomas (RPSs) located in the lower abdominal quadrants involving iliac vessels are difficult to manage. This study introduced a 5-step method for en bloc resection with graft interposition using the abdominoinguinal approach and evaluated its efficacy and safety.

**Methods:**

Data of 24 consecutive patients who met the inclusion criteria from 272 patients with RPS who underwent surgical treatment between April 2015 and April 2022 were retrospectively collected and analyzed.

**Results:**

The patients underwent left- or right-sided abdominoinguinal incision. In all patients, the abdominoinguinal approach provided good exposure, and complete resection was achieved. Iliac artery+vein, vein, and artery resection and replacement by graft were performed in 70.8%, 25.0%, and 4.2% of patients, respectively. Additional resected organs mainly included the colon, ureter, bladder, kidney, and abdominal wall. The median number of organs resected was 5. In 37.5% of patients, reconstruction of the lower abdominal wall and inguinal ligament was performed using a mesh. Venous graft thrombosis occurred in 21.7% of patients, while no patient had pulmonary embolism or arterial occlusion. Major complications occurred in 20.8% of patients, and no 30-day mortality was observed. The estimated 5-year local recurrence and distant metastasis rates were 54.4% and 22.1%, respectively, with a median recurrence-free survival of 27 months.

**Conclusions:**

En bloc resection of RPS involving iliac vessels with graft interposition using the abdominoinguinal approach is feasible and advantageous. Good complete resection rate and safety can be achieved. The long-term survival benefit of this surgical approach should be verified by further large-scale prospective controlled studies.

## 1 Introduction

Retroperitoneal sarcomas (RPSs) are rare malignant tumors with numerous heterogeneous histological subtypes (1). Surgery currently remains the only potentially curative treatment to achieve local control. Local recurrence (LR) is the leading cause of death in most patients (2, 3). Therefore, to minimize the LR, complete en bloc gross resection with negative margins is recommended in the surgical management of RPS (4).

However, surgery with satisfactory margins for RPS is challenging. Because of their large size and involvement of adjacent critical structures, RPS resection commonly necessitates multivisceral resection (MVR), including resection and reconstruction of major vessels such as the inferior vena cava (IVC), iliac artery, and iliac vein (5, 6).

Depending on tumor location, size, and aggressiveness, different surgical approaches are used to achieve complete resection. Most RPSs can be approached and resected *via* a midline incision. However, for a subset of patients with RPS, tumors in the lower abdominal quadrants extend laterally and obscure iliac vessels or are located in the iliac fossa and extend inferiorly toward the inguinal ligament. To achieve en bloc resection, the involved iliac vessels should be resected en bloc with the tumor to obtain a clear margin. In such cases, midline incision restricts the exposure of the caudal or lateral aspects of the tumors, making resection and anastomosis difficult.

We found that the abdominoinguinal approach could provide a solution (7). Dividing the anterolateral abdominal wall muscles and inguinal ligament opens up the space and facilitates segmental resection and anastomosis of the iliac vessels and en bloc removal of the tumor. This study aimed to describe in detail the abdominoinguinal approach in en bloc resection of lateral pelvic RPS involving iliac vessels with graft interposition and analyze the short- and long-term outcomes.

## 2 Methods

### 2.1 Patients

This study was approved by the Ethics Committee of Peking University Cancer Hospital and performed according to the 1975 Helsinki Declaration and its later amendments or comparable ethical standards. All patients provided written informed consent before surgery for the use of their anonymized data.

Between April 2015 and April 2022, 272 consecutive patients underwent RPS-related surgery at the Peking University Cancer Hospital Sarcoma Center. Patients could be grouped into primary and recurrent cohorts, and the treatment algorithm and indications of surgery for both cohorts have been previously described (5). The inclusion criteria of this study were as follows: resection *via* the abdominoinguinal approach was performed; iliac vessels (artery, vein, or both) were resected en bloc with the tumor; the graft was interposed to perform reconstruction; and the pathology result was RPS according to the 2020 World Health Organization criteria for soft tissue tumors (1). Twenty-four patients met the inclusion criteria.

**Figure 1:**
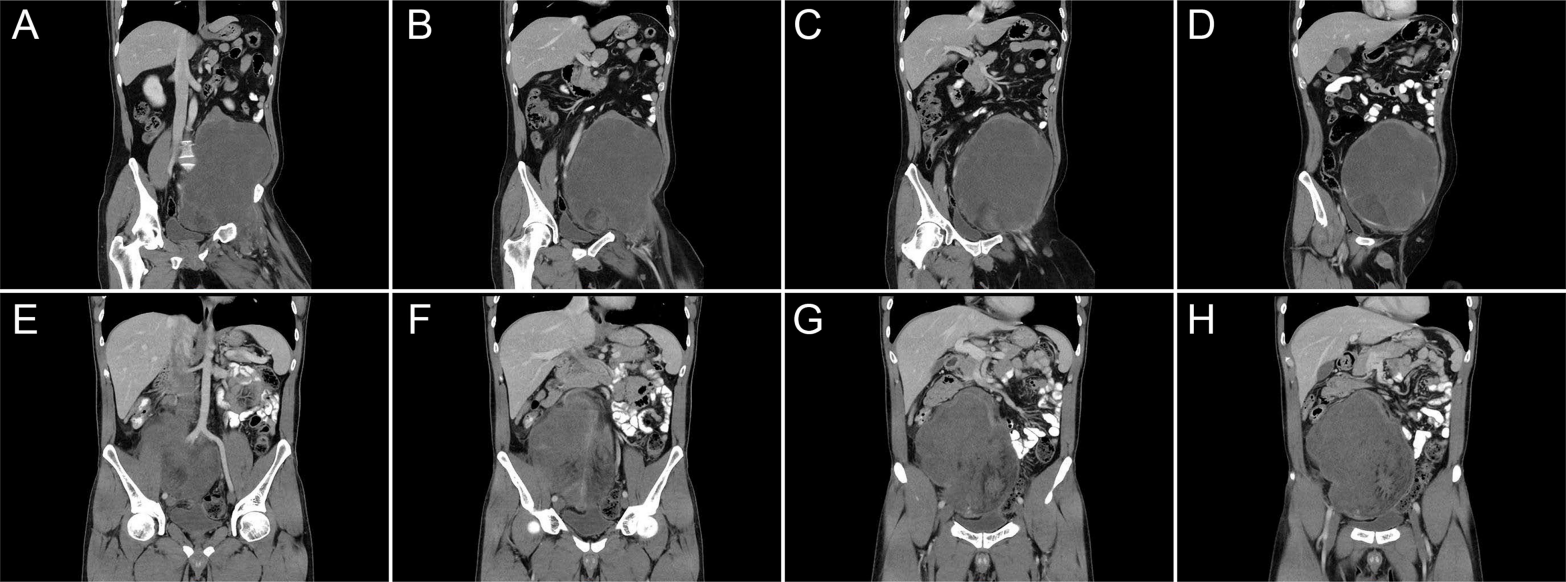
Representative imaging of retroperitoneal sarcoma in the left **(A–D)** and right **(E–H)** lower quadrants of the abdomen, which extend laterally and obscure the iliac vessels.

All data were retrieved from the prospectively collected sarcoma database of our center and were retrospectively analyzed. The extent of iliac vessel involvement was evaluated using preoperative abdominopelvic contrast-enhanced computed tomography (CT) or magnetic resonance imaging (MRI) (**Figure 1**). All patients underwent surgical treatment by the same surgical team led by CY Hao. The team has considerable expertise in RPS-related surgery and is capable of performing major abdominal operations including the resection and reconstruction of major vessels (such as the aorta, IVC, and iliac vessels). Surgical resections were classified as macroscopically complete (R0/R1) or incomplete (R2) in accordance with most previous studies evaluating prognostic factors for RPS, because the large surface area and anatomic location of the RPS make it difficult to perform a reliable microscopic assessment of margins (2, 3, 5, 6, 8). Tumor grading was determined by the 3-tiered grading system of the Fédération Nationale des Centres de Lutte Contre le Cancer criteria (9). Complications were recorded and graded using the Clavien–Dindo classification and considered “major” if grade III or higher was observed (10).

### 2.2 Surgical procedure

#### 2.2.1 Step 1: Abdominoinguinal incision and field exposure

**Figure 2:**
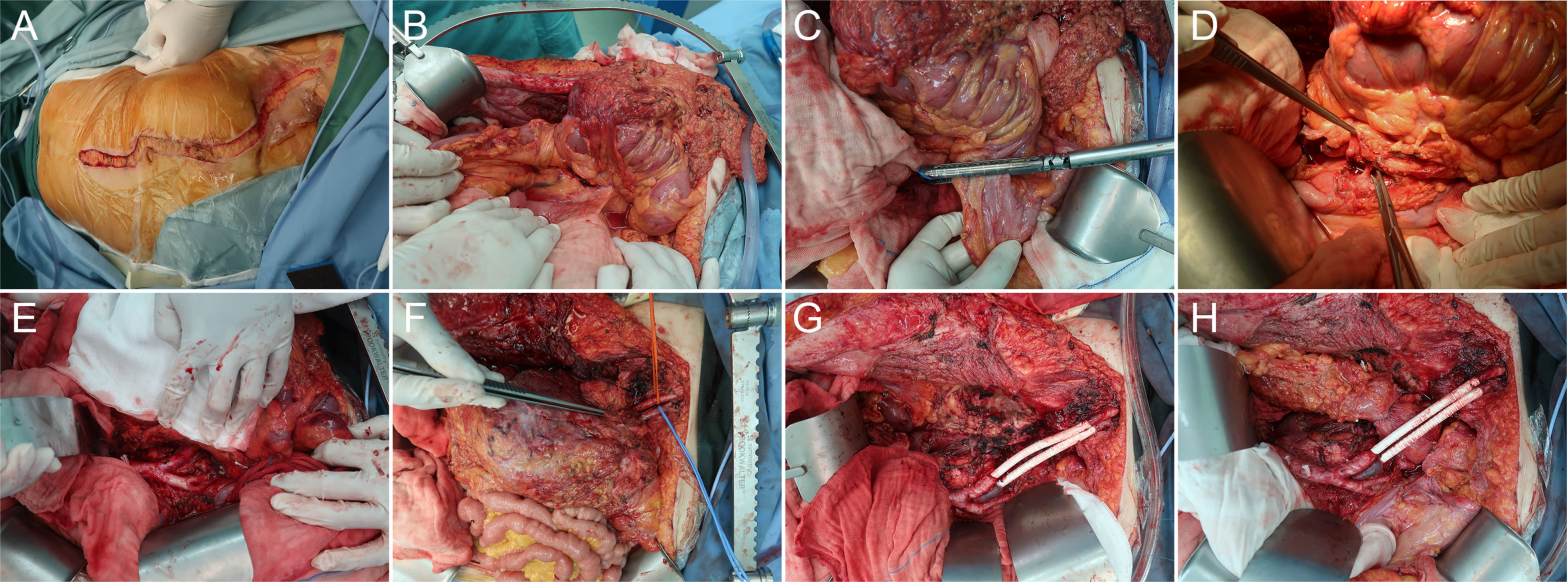
Representative case of en bloc resection of retroperitoneal sarcoma encasing the left iliac vessels with graft interposition *via* the abdominoinguinal approach. **(A)** A left-sided abdominoinguinal incision is made from approximately 5 cm above the umbilicus to approximately 2 cm above the pubic symphysis, then from the lower end point to the left mid-inguinal point, and then vertically into the femoral triangle. **(B)** The entire lower abdominal wall could be laterally retracted and full access to the abdomino-pelvic organs is acquired. **(C)** The infiltrated sigmoid is transected by GIA. **(D)** The root of the inferior mesenteric artery is dissected. **(E)** The left common iliac artery and vein are dissected from the bifurcation of the aorta and the apex of the triangle of Marcille to the point of involvement by the tumor. **(F)** The left external iliac artery and vein are dissected in the groin, released by dividing branches and tributaries, and lifted with vessel loops distal to the point of involvement. **(G)** After removing the tumor, the en bloc resected iliac artery and vein are replaced with a graft (polytetrafluoroethylene [PTFE] with integrated rings, 8 mm in diameter) with a 5-0 Prolene running suture. **(H)** Anastomosis of the descending colon to the rectum is performed.

First, a midline incision was made from above the umbilicus to approximately 2 cm above the pubic symphysis. The midline incision length depended on the tumor size and planned resection range. If necessary, the midline incision could be extended upward to the xiphoid. Subsequently, the incision was extended transversely from the lower endpoint to the right or left mid-inguinal point and then vertically into the femoral triangle (**Figure 2A**). In this step, the femoral pulse can be touched, and the incision should be just over the femoral artery and oriented in its direction. The rectus abdominis was transected, inguinal ligament was divided, entire lower abdominal wall was laterally retracted, and full access to the abdominopelvic organs was acquired (**Figure 2B**). Using this approach, the iliac vessels in the pelvis and femoral vessels in the groin could be exposed in one continuous field; thus, it was possible to control the vessels proximally and distally.

#### 2.2.2 Step 2: Dividing other involving structures

Before transecting the iliac vessels, the other involved structures were divided in advance, if possible (**Figures 2C, D**). The area difficult to expose in this step was left until the iliac vessels were transected and the tumor was further mobilized. For recurrent cases, we usually had to first divide the adhesions and mobilize the small bowel loops from the pelvis. The tumor is commonly fixed to the pelvic wall, and the anterolateral parietal peritoneum and abdominal wall muscles need to be resected together. Sometimes, the bladder, rectosigmoid or ileocecum, and ureter can be mobilized and separated to expose the retroperitoneum. However, more often, one or more such organs were involved in the tumor and had to be resected en bloc and reconstructed. Total pelvic exenteration was seldom observed in this subset of patients.

#### 2.2.3 Step 3: Transection of proximal and distal iliac vessels

After exposure of the retroperitoneum, the common and external iliac arteries and veins were dissected. The dissection started proximally at the bifurcation of the aorta and IVC and the apex of the triangle of Marcille and proceeded distally to the point of involvement by the tumor (**Figure 2E**). The iliac vessels were released and encircled with vessel loops proximal to the point. Similarly, the external iliac (femoral) vessels were dissected in the groin, released by dividing branches and tributaries, and lifted with vessel loops distal to the point of involvement (**Figure 2F**). Subsequently, the involved iliac vessels were transected. If the origin was involved, internal iliac vessels were also transected. In this step, sometimes the common and external iliac artery could be separated from the tumor over the entire length to avoid transection and reconstruction, while the iliac vein could seldom be separated.

#### 2.2.4 Step 4: Tumor removal and graft interposition

After transecting the iliac vessels, the potential tumor mobility dramatically improved. Mobilization and gentle retraction of the tumor were then possible, providing the operator access to the lateral and posterior planes of dissection. After dividing the remaining involved muscles and nerves, the tumor was removed en bloc. Sometimes, there was no room to divide the internal iliac vessels until this step, and of special note was the bleeding by tearing of the internal iliac vessels or their branches and tributaries. The use of endo-GIA to secure this division should be considered. The resected iliac vessels were replaced with a graft (polytetrafluoroethylene [PTFE] with integrated rings, usually 8 mm in diameter) with a 5-0 Prolene running suture (**Figure 2G**).

#### 2.2.5 Step 5: Other reconstructions

**Figure 3:**
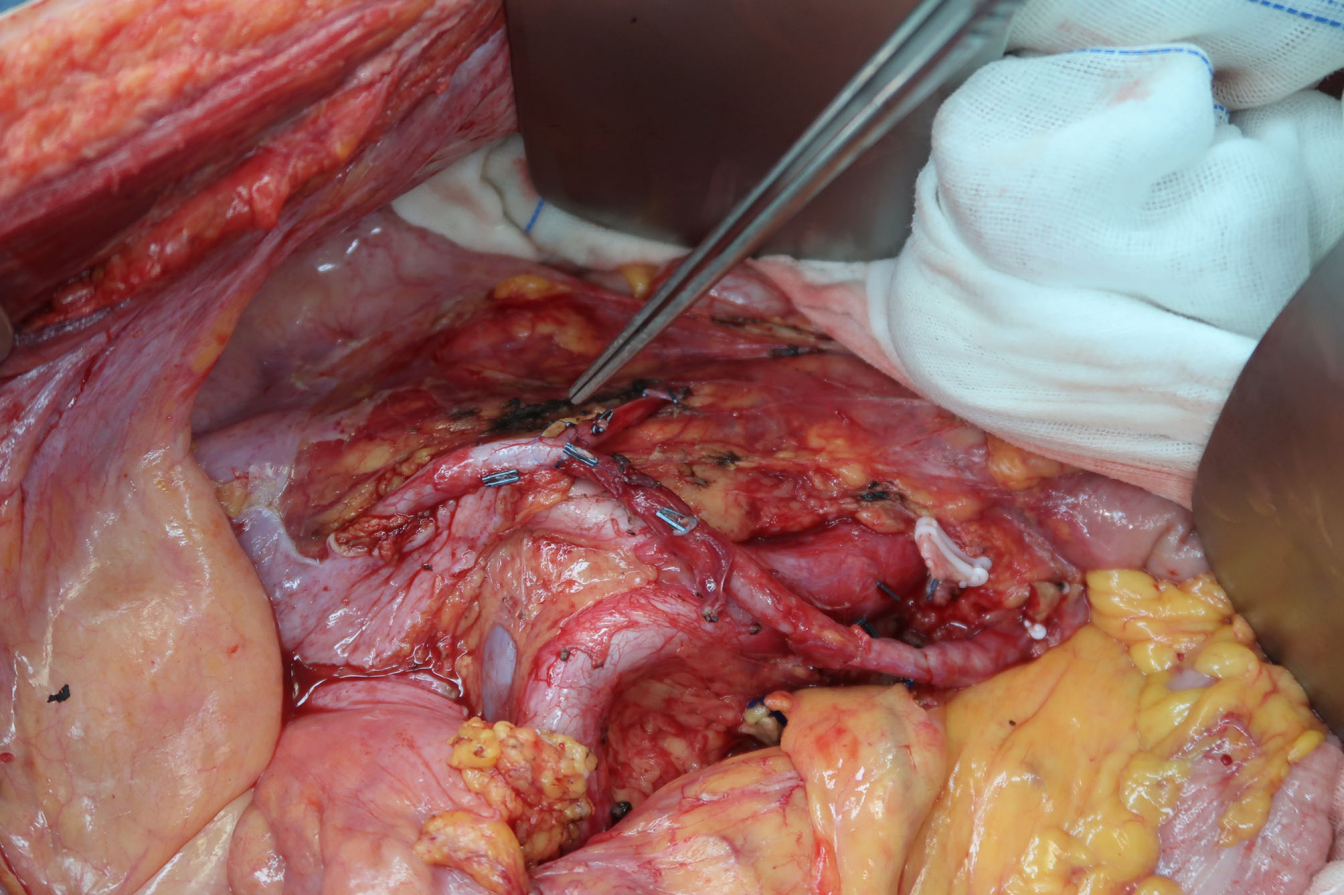
Anastomosis of the end of the resected left ureter to the side of the right ureter.

If the tumor invaded the rectosigmoid or ileocecum, segmental resection and end-to-side or end-to-end anastomosis were performed (**Figure 2H**). Protective ileostomy was considered when the operator judged the risk was high. If a modest portion of the bladder was involved and partially resected, it was possible to directly repair the defect. However, cystectomy and an ileal conduit could be safe choices if a significant portion of the bladder was resected. Ureteral reconstruction mainly depended on the location and extent of the defect. End-to-end ureteral anastomosis, ureterovesical replantation, and use of an isolated segment of ileum anastomosed to the proximal end of the ureter and distally to the bladder are common options. In addition, anastomosis of the end of the resected ureter to the side of the contralateral ureter was also an alternative (**Figure 3**). If the anterior abdominal wall, including the inguinal ligament, is involved and resected with a significant defect, reconstruction can be performed using a biologic mesh.

### 2.3 Postoperative management

According to the general principles of surgical and supportive care, standard postoperative treatments were administered, including fluid balance, adequate replacement of electrolytes, prophylactic anti-infection, and total parenteral nutrition. Barring special circumstances, subcutaneous low-molecular weight heparin (LMWH) with intermittent compressive devices were routinely used 12 hours after surgery. Generally, patients were encouraged to stand and walk around the bed using a walker within 48 hours after surgery. All patients underwent abdominopelvic contrast-enhanced CT and lower- extremity vascular ultrasound before discharge. Oral anticoagulants (such as Warfarin and Rivaroxaban) were administered to patients until 6 months after discharge. For patients undergoing iliac artery reconstruction or patients with thrombocytosis, anti-platelet agent (Aspirin) was also administered simultaneously. Patients were routinely followed up by clinical examination, laboratory tests, and CT or MRI every 3–4 months for 2 years, every 6 months for 3 years, and yearly thereafter.

### 2.4 Statistical analysis

The primary outcomes were complete (R0/R1) resection rate, major complication rate and 30-day mortality. The secondary outcomes were 5-year recurrence-free survival (RFS), 5-year LR, and 5-year distant metastasis (DM) rates. LR was defined as all recurrent lesions located in the retroperitoneum or abdominopelvic cavity. DM was defined as new lesions located at sites other than the retroperitoneum or abdominopelvic cavity, such as the liver, lungs, and spine. RFS was calculated from the date of surgery to the date of LR/DM or death, whichever was first observed. Standard descriptive statistics were calculated for categorical (i.e., frequency and percentage) and continuous (i.e., median and range) data, as listed in the tables. The survival curve was estimated using the Kaplan–Meier method and compared using the log-rank test. Results were considered statistically significant if a two-sided *P*-value <0.05 was achieved. Statistical analyses were performed using SPSS version 26.0 (SPSS Inc., Chicago, IL, USA).

## 3 Results

### 3.1 Clinicopathological and operative characteristics

The clinicopathological characteristics of the patients are shown in **Table 1**. A total of 24 patients (13 men and 11 women; median age 55 years; range, 30-72 years) were included in this study. Of these patients, 19 (79.2%) and 5 (20.8%) were identified as primary and recurrent patients, respectively. Among the 5 patients with recurrent disease, four patients experienced once RPS-related surgery before and one patient experienced twice. The predominant histologic subtypes were dedifferentiated liposarcoma and leiomyosarcoma, followed by well-differentiated liposarcoma. Lower limb symptoms, including pain, edema, and paresthesia, were present in 8/24 (33.3%) patients before surgery. Among 24 patients, two of them received Eribulin plus Anlotinib plus Camrelizumab treatment before surgery, and both had stable disease according to the Response Evaluation Criteria in Solid Tumors v.1.1 (RECIST v1.1). None of the rest patients underwent preoperative systemic or radiation therapy.

**Table 1:** Clinicopathological characteristics of patients.

Parameter	Patients (n=24)
Sex ratio (Male/Female)	13/11
Age (years)*	55 (30–72)
Presentation status	
Primary RPS	19 (79.2%)
Recurrent RPS	5 (20.8%)
Lower limb symptoms†	
Yes	8 (33.3%)
No	16 (66.7%)
Tumor size (cm)*	20 (9−45)
Pathological subtypes	
Dedifferentiated liposarcoma	9 (37.5%)
Leiomyosarcoma	8 (33.2%)
Well-differentiated liposarcoma	3 (12.5%)
Pleomorphic liposarcoma	1 (4.2%)
Myxoid liposarcoma	1 (4.2%)
Undefferentiated pleomorphic sarcoma	1 (4.2%)
Rhabdomyosarcoma	1 (4.2%)
FNCLCC grade	
1	3 (12.5%)
2	10 (41.7%)
3	11 (45.8%)
Preoperative treatment	2 (8.3%)
Eribulin plus Anlotinib plus Camrelizumab	2 (8.3%)
Radiation therapy	0
Adjuvant treatment	0

*Median with range. † includes pain, edema, and paresthesia. RPS, retroperitoneal sarcoma; FNCLCC, Fédération Nationale des Centres de Lutte Contre le Cancer.

Operative information and short-term postoperative outcomes are presented in **Table 2**. Left–sided abdominoinguinal incisions were made in half of the patients, and right-sided in the other half. The abdominoinguinal approach provided good exposure in all 24 patients, and R0/R1 resection was achieved in each patient. In 17/24 (70.8%) patients, both the iliac artery and vein were involved and replaced by grafts. Six of 24 patients (25%) underwent only vein resection, and 1 patient (4.2%) underwent only artery segmental resection and graft interposition. All patients underwent MVR, and the median number of resected organs was 5. Common additional resected organs included the colon, ureter, bladder, kidney, abdominal wall (including the inguinal ligament), and surrounding muscles and nerves. Distal pancreatectomy, splenectomy, hysterectomy, and salpingo-oophorectomy were also performed.

**Table 2:** Operative characteristics and postoperative complications of patients.

Parameter	Patients (n=24)
Operative time (min)*	472 (327−767)
Estimated blood loss (ml)*	2100 (1000−5300)
Side of abdominoinguinal incision	
Left	12 (50%)
Right	12 (50%)
Involving iliac vessels	
Artery + vein	17 (70.8%)
Vein only	6 (25.0%)
Artery only	1 (4.2%)
No. of resected organs*	5 (3−11)
Main additional resection	
Segmental colectomy	20 (83.3%)
Nephrectomy	14 (58.3%)
Segmental ureterectomy	8 (33.3%)
Cystectomy (Partial or total)	8 (33.3%)
Resection of partial abdominal wall & inguinal ligament	9 (37.5%)
Completeness of resection	
R0/R1	24 (100%)
R2	0 (0%)
Lifelong ostomy	3 (12.5%)
Ileal conduit	2 (8.3%)
Colostomy	1 (4.2%)
Major complications	5 (20.8%)
Arterial anastomotic bleeding	1 (4.2%)
Abdominal bleeding	1 (4.2%)
Abdominopelvic/incision infection	3 (12.5%)
Acute kidney insufficiency	1 (4.2%)
Deep venous thrombosis	1 (4.2%)
Postoperative hospital stay (days)*	18 (10−43)
30-day mortality	0

*Median with range.

Regarding reconstruction, six patients underwent partial cystectomy and direct suture repair and two underwent total cystectomy and ileal conduit. Other patients undergoing segmental ureterectomy included: 1 underwent end-to-end anastomosis; 2 underwent ureterovesical replantation; 1 underwent ileal replacement of the partial ureter; 2 underwent anastomosis of the end of the resected ureter to the side of the contralateral ureter. Moreover, one patient underewent lifelong colostomy. In 9/24 patients (37.5%), reconstruction of the lower abdominal wall and inguinal ligament was performed using a mesh.

### 3.2 Safety and complications

**Figure 4:**
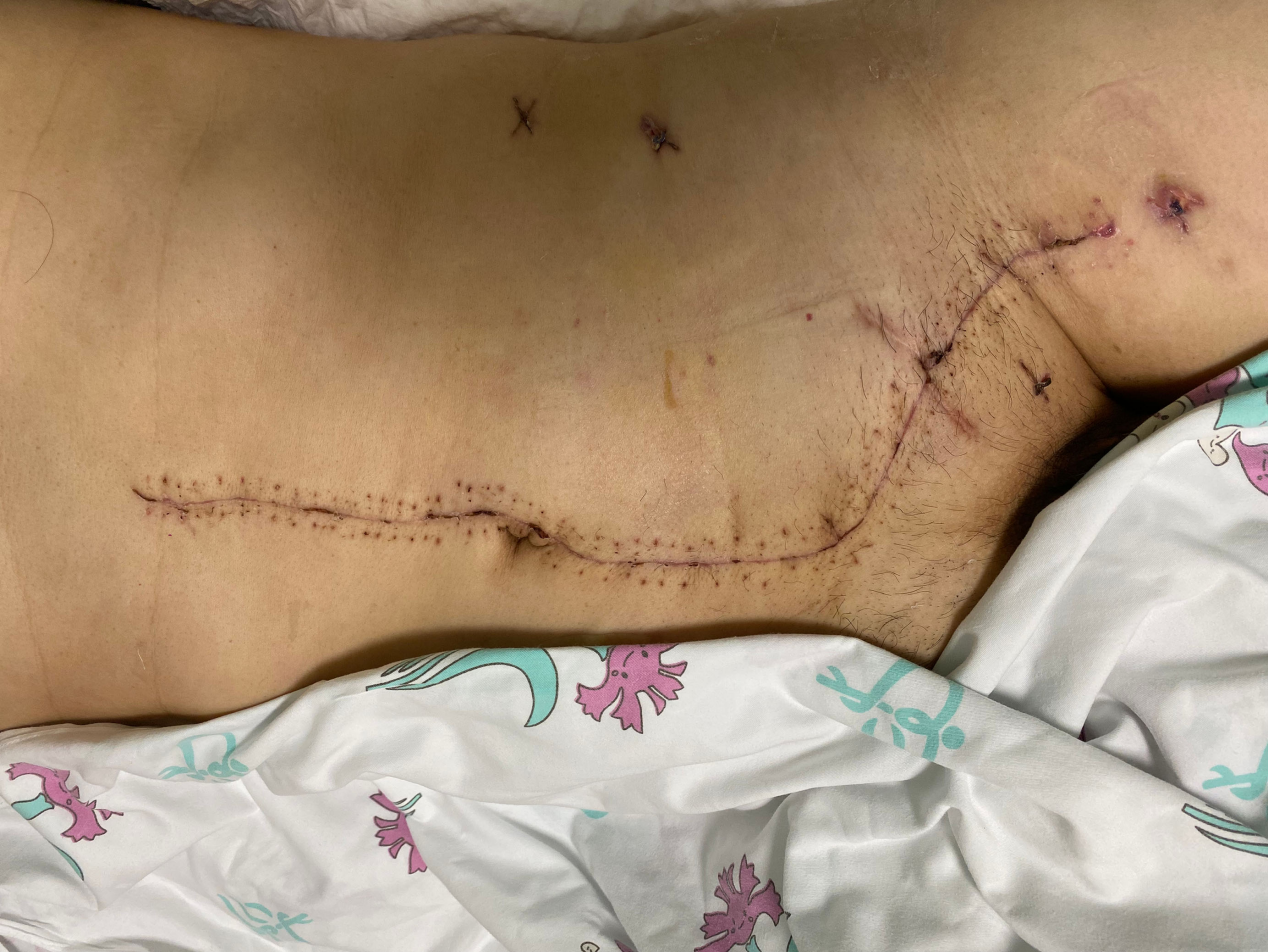
The well healed left abdominoinguinal incision.

The abdominoinguinal incision generally healed well (**Figure 4**), and severe incision infection occurred in 2/24 patients (8.3%). Postoperative venous graft thrombosis/deep venous thrombosis (DVT) was found in 5/23 patients (21.7%), while IVC filter was required in only 1 patient. In other patients, DVT decreased or disappeared after treatment with LMWH. Pulmonary embolism or arterial occlusion was not observed in any patient. Postoperative neurological impairment of the ipsilateral lower limb, including weakness, pain, or numbness, was found in 8/24 patients (33.3%). Major postoperative complications (Clavien–Dindo classification ≥III) occurred in 5/24 patients (20.8%) (**Table 2**). Among them, reoperation was required in 2/24 patients (8.3%) because of arterial anastomotic bleeding secondary to abdominopelvic and incision infection and pancreatic stump bleeding secondary to pancreatic fistula and abdominopelvic infection, respectively. The other 3 major complications included incision infection, acute kidney insufficiency, and DVT. No 30-day mortality was observed, and one patient required a second hospital admission because of incision infection.

### 3.3 Survival

None of the patients received adjuvant systemic or radiation therapy. The median follow-up was 18.0 (95% confidence interval 2.1–33.9) months. Overall, 9 patients developed LR disease, 3 had DM, and 10 died during follow-up. The estimated 5-year LR, DM and RFS rates of all the 24 patients were 54.4%, 22.1% and 45.6%, respectively, with an estimated median RFS (mRFS) of 27 months.

Further analysis was performed according to presentation status. The estimated 5-year LR, DM and RFS rates between patients with primary and recurrent RPS were 53.9% vs. 60.0%, 21.4% vs. 20.0% and 46% vs. 40%, respectively. With an mRFS of 27 months, the patients with primary RPS presented a better RFS trend in contrast with that of 3 months in the recurrent group, although significance was not reached likely due to the low volume of patients (*P* = 0.13).

**Figure 5:**
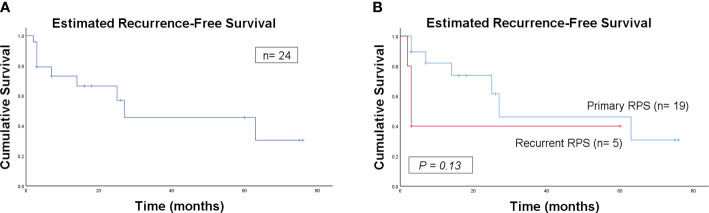
Kaplan–Meier curve of estimated recurrence-free survival for all the patients **(A)** and for comparison of primary and recurrent patients **(B)**.

The estimated RFS curves for the patients are shown in **Figure 5**.

## 4 Discussion

The abdominoinguinal incision was initially described by Karakousis (11–13). The present study reported 24 patients who underwent en bloc resection of RPS involving iliac vessels with graft interposition using the abdominoinguinal approach, which may be the largest single-center case series in the English literature to date. We describe in detail a 5-step method used by our team over the last 7 years. It has been a standardized approach for RPS in the lower abdominal quadrants for resection and reconstruction of iliac vessels at our center.

Based on our experience, the biggest merit of this approach is that it opens up space and allows operation under direct vision. The iliac vessels on the side of involvement in their continuity with the femoral vessels in the groin can be exposed in one continuous field, which facilitates satisfactory resection with negative margins as well as graft interposition. In addition, the involved lateral and anterior abdominal walls could be resected en bloc. In fact, R0/R1 resection was achieved in all patients included in this study. The repair of defects of the lower abdominal wall and inguinal ligament by biological mesh was needed in 9/24 (37.5%) of the patients in our study. The abdominoinguinal incision generally healed well, with an acceptable major infection rate of 8.3%.

RPS involving the major venous or arterial vessels is challenging for surgeons. Resection of RPS with major vessels reconstruction was reported with a 36%-38% major complication rate and 4%-6% of mortality, higher than those without vascular reconstruction (14, 15). The rate of resection of major vessels in high-volume sarcoma centers is approximately 10–26% (2, 5, 15, 16), and iliac vessels occupy a large portion. Either originating from them or secondarily encasing or invading them is an indication for en bloc resection. In our center, to minimize microscopically positive margins, we generally tend to resect potentially involved blood vessels en bloc with the tumor rather than attempt to dissect them under the adventitia if there is a chance of complete resection.

Technically, to achieve satisfactory resection and anastomosis, complete dissection and mobilization of the proximal and distal iliac vessels are key. It is more likely to be achieved *via* the abdominoinguinal approach than *via* the midline approach. Theoretically, it is best to perform the transection of vessels as the last step before removing the tumor so that the cross-clamp time can be as short as possible. However, based on our experience, sometimes unless the iliac vessels are transected, no room can be created to retract the tumor and access the lateral and posterior planes. Therefore, the cross-clamp time varied on different cases. In fact, it is not so strict because even 2–3 hours of complete occlusion of the iliac vessels will not result in irreversible ischemia or dysfunction of the lower limb.

In cases of short vascular resection, direct end-to-end anastomosis is both feasible and safe. However, this rarely occurs in this subset of patients. Therefore, the PTFE graft was routinely used to perform long vascular reconstruction in our center. There is no doubt about the need for arterial reconstruction. In 18 patients who underwent segmental iliac artery resection and graft interposition, no postoperative lower limb critical ischemia caused by occlusion occurred. However, the need for vein replacement is controversial mainly because of the risk of developing graft thrombosis and subsequent pulmonary embolism (17, 18). In our center, we attempted to reconstruct the iliac vein as long as possible to diminish severe lower limb edema and improve functional outcomes. The incidence of venous graft thrombosis in this series was 21.7% (5/23), most of which (4/5) could be controlled by treatment with LMWH, and no cases of pulmonary embolism were found. These results are comparable to reported data from other high-volume centers (15, 19) and at least not against our policy of vein reconstruction.

Resecting major vessels combined with the digestive tract, especially the colorectum with graft interposition, is regarded as relatively contraindicated because it is considered to greatly increase the risk of critical infection. However, it is unavoidable in RPS-related surgeries because of its infiltration tendency (20). The development of surgical instruments, techniques, and perioperative management has increased the safety of combination resection (15). Our study results also indicate that the safety of such an MVR is acceptable. Serious infections occurred in only 3 patients (12.5%), although 2 of them resulted in secondary hemorrhage and reoperation. Optimal perioperative management (including preoperative nutritional support, bowel preparation, and prophylactic anti-infection), appropriate methods of resection and reconstruction, thorough irrigation with saline before abdominal closure, and effective drainage may be key factors. In addition, protective ileostomy may be worthy of consideration as the lesser of two evils.

Postoperative neurological impairment of the lower limb after MVR is another issue of concern for RPS. Callegaro et al. (21) reported that 76% of patients had some degree of neuropathy and it occurred more frequently in patients who underwent complete or partial resection of the psoas muscle. Fiore et al. (22) reported that neuropathic pain was found in 41.4% of the patients and was significantly associated with resection of the psoas muscle. In this series, one-third of patients experienced various degree of weakness, pain, or numbness of the lower limb after surgery, which interfered with patients’ quality of life for a relatively long time. However, owing to the location, extent, and anatomic relationship of tumors, it is sometimes inevitable for this subset of patients, especially considering that accompanying symptoms such as pain, edema, and paresthesia of the lower limb were present in 33.3% of patients before surgery. Surgeons should balance completeness and preservation of organ function during surgery. However, when R0/R1 resection was possible, we performed as radical a surgery as necessary to reduce the recurrence rate and remove surrounding infiltrated muscles and nerves en bloc with the tumor and major vessels.

The estimated 5-year LR, DM, and RFS rates in this study were 54.4%, 22.1%, and 45.6%, respectively, with an mRFS of 27 months. These data were similar to those reported by Wortmann et al. (23), with a 2-year RFS of 46% and an mRFS of 23 months in the RPS subgroup who underwent major vessel resection and reconstruction (n=20, 12 for primary and 8 for recurrent RPS). Radaelli et al. (19) reported a lower LR rate (12%) and a higher DM rate (58%). However, the data were analyzed in patients with sarcoma in both extremities and the retroperitoneum, in which RPS accounted for only 41%. Our previous study (5) based on 242 patients (145 primary and 97 recurrent RPS) also showed a comparable result. The estimated 5-year LR, DM, and RFS rates of all patients were 56.1%, 18.9%, and 40.0%, respectively, with an mRFS of 32 months. These results above may indicate that encasement of the iliac vessels does not represent a contraindication to surgery, and en bloc resection with a clear margin can achieve a comparable RFS for this subset of patients. However, the long-term survival benefit of this surgical approach should be verified by further large-scale prospective controlled studies.

Notably, owing to the rarity and complexity of this subset of patients, the judgment of indications and the preferred surgical policy may vary widely among different institutions, especially for patients with recurrent RPS. This is a significant reason why patient management by experienced multidisciplinary team (MDT) in a specialized sarcoma center is strongly recommended (24, 25). As one of the largest specialized RPS centers in China, we built a stable experienced MDT focusing on RPS. The indications and patient selection for surgery at our center were based on the treatment algorithm established during the long-term collaboration (5). Imaging evaluation (the chances of performing R0/R1 resection and the status of distant metastasis), tumor biology (including pathological subtypes, multifocality, disease-free interval, and response to systemic therapy), and symptoms (bleeding, obstruction, and intolerable abdominal distension and pain) are major factors that influence the decision-making. Due to the unsatisfactory effect of radiation therapy and chemotherapy observed in most RPS cases, we adopted an aggressive surgical strategy for potentially resectable tumors, even for the patients with recurrence. In this study, among the 5 patients (20.8%) with recurrent disease, four patients experienced once RPS-related surgery before and one patient experienced twice. Given that the tumors of these 5 patients were regarded as resectable, the decision of surgery was made by MDT members based on various disease- and patient-specific factors.

This study had certain limitations. First, it was a retrospective cohort study conducted at a single institution with a small sample size, which was lack of a control collective. Second, because of the retrospective nature, it did not report on some intraoperative details such as the cross-clamp time of each case. Third, the follow-up time for some patients was quite short. Last, enrolling both primary and recurrent RPS patients with different histological subtypes is a major confounding factor in the survival analysis. However, considering the rarity and complexity of this subset of patients, it is challenging for surgeons to conduct prospective randomized controlled trials with large sample sizes. This preliminary study introduced a standardized 5-step method at our center and presented encouraging results. The study findings could be helpful in improving clinical decision-making and treatment in similar settings.

## 5 Conclusions

En bloc resection of RPS involving iliac vessels with graft interposition using the abdominoinguinal approach is feasible and advantageous. Good complete resection rate and safety can be achieved. The long-term survival benefit of this surgical approach should be verified by further large-scale prospective controlled studies.

## Data availability statement

The raw data supporting the conclusions of this article will be made available by the authors, without undue reservation.

## Ethics statement

The studies involving human participants were reviewed and approved by Ethics Committee of Peking University Cancer Hospital. The patients/participants provided their written informed consent to participate in this study. Written informed consent was obtained from the individual(s) for the publication of any potentially identifiable images or data included in this article.

## Author contributions

AL and C-YH contributed to the conception and design of the study. AL, B-NL, D-NL and ZW collected, analyzed, and interpreted the patient data. AL wrote the first draft of the manuscript. B-NL, D-NL, and ZW wrote the sections of the manuscript. All authors contributed to the article and approved the submitted version.
